# Toward Closed-Loop Electrical Stimulation of Neuronal Systems: A Review

**DOI:** 10.1089/bioe.2020.0028

**Published:** 2020-12-16

**Authors:** Jarno M.A. Tanskanen, Annika Ahtiainen, Jari A.K. Hyttinen

**Affiliations:** BioMediTech Institute and Faculty of Medicine and Health Technology, Tampere University, Tampere, Finland.

**Keywords:** brain, closed-loop, electrical stimulation, neuronal cells, neuronal stimulation, open-loop

## Abstract

Biological neuronal cells communicate using neurochemistry and electrical signals. The same phenomena also allow us to probe and manipulate neuronal systems and communicate with them. Neuronal system malfunctions cause a multitude of symptoms and functional deficiencies that can be assessed and sometimes alleviated by electrical stimulation. Our working hypothesis is that real-time closed-loop full-duplex measurement and stimulation paradigms can provide more in-depth insight into neuronal networks and enhance our capability to control diseases of the nervous system. In this study, we review extracellular electrical stimulation methods used in *in vivo*, *in vitro*, and *in silico* neuroscience research and in the clinic (excluding methods mainly aimed at neuronal growth and other similar effects) and highlight the potential of closed-loop measurement and stimulation systems. A multitude of electrical stimulation and measurement-based methods are widely used in research and the clinic. Closed-loop methods have been proposed, and some are used in the clinic. However, closed-loop systems utilizing more complex measurement analysis and adaptive stimulation systems, such as artificial intelligence systems connected to biological neuronal systems, do not yet exist. Our review promotes the research and development of intelligent paradigms aimed at meaningful communications between neuronal and information and communications technology systems, “dialogical paradigms,” which have the potential to take neuroscience and clinical methods to a new level.

## Introduction

Networking and electrical activity are the hallmarks of neuronal cells.^1,2^ Neuronal activity is the basis of actions and thoughts of all more evolved life-forms and can be stimulated by perceived sensory input or other external stimuli, many types of which have been used both clinically^3^ and experimentally. The present review concentrates on extracellular electrical stimulation^i^ (EES) and touches on associated extracellular electrical measurement (EEM) modalities that can be used to study neuronal cell and network structures, properties, and functions *in vivo* (in a living creature), *in vitro* (in a dish), *in silico* (in a computational simulation), and in clinical applications. The primary purpose of this review is to provide a broad view of the emerging possibilities of closed-loop^4^ EEM-EES control of biological neuronal systems.

In the introduction, the rationale for closed-loop EEM-EES systems *in vivo*, *in vitro*, and *in silico* is outlined, and thereafter, two different views are offered on how such systems can be classified. Next is an introduction to select prerequisites of closed-loop EEM-EES systems. The two main prerequisites are that the (1) computational system is adequately faster than the biological phenomena to be controlled and (2) target neuronal system is controllable by the selected controller. In this study, the controller is the part of the closed-loop system that manages the operation of the stimulator based on the measurements. Introduction to neuronal cells and systems and stimulation modalities is then given in a separate introductory section. The actual review presents EES methods for neuronal systems *in vivo*, *in vitro*, and *in silico* in separate subsections. Finally, the discussion and conclusions overview a couple of often overlooked aspects and note that controlling complex dynamic neuronal systems calls for adaptive closed-loop EEM-EES systems.

### The rationale of closed-loop EEM-EES systems

The rationale for the present work is summarized in the working hypothesis that real-time closed-loop signal analysis and stimulation, which continuously adapt to the observed neuronal system function or state (in a trackable way) may offer more in-depth insights into the workings of neuronal systems and greater opportunities for medical remedies. Biological neuronal circuits in a living creature are in constant interaction with other neuronal circuits, or the creature's input/output, such as sensory inputs and language and movement outputs. Thus, adequately designed real-time closed-loop EEM-EES systems could provide improved biomimicking systems *in vitro*.

This review also aims to promote the research and development of real-time closed-loop paradigms that intend to “discuss” with the neuronal systems or “interrogate” them. We call such methods “dialogical paradigms” and “dialogical algorithms,” since there would effectively be “a dialog” between the neuronal and computational systems. Neuronal action potentials (APs) and higher brain functions are orders of magnitude slower than the basic operations of information and communications technology (ICT) components in EEM-EES systems. Basic EEM processing is also feasible within the time frame of AP activity. Thus, real-time closed-loop EEM-EES systems are a feasible opportunity.

This review serves as a primary survey for electrical extracellular open-loop versus closed-loop stimulation systems. Closed-loop systems that utilize other stimulation modalities than electrical stimulation can be constructed similarly. To that end, several stimulation modalities are also briefly overviewed in this review. One goal is to raise interest in developing more computationally advanced dialogical systems and advocate the significant research question on how closed-loop EES could open new avenues for better control of neuronal systems, with impacts from basic research to clinical applications.

The advancement of both *in vivo* and *in vitro* closed-loop EEM-EES is valuable from both clinical and neuroscientific points of view. As seen above, *in vivo* systems are used in nervous system disease and symptom control and as medical aids employing brain–computer interfaces^5^ (BCIs). There is no reason why future developments should not bring further benefits to patients in the form of more reliable, safer, and easier to use remedies and aids that exhibit higher treatment efficacy. At the same time, these patients and EEM-EES technologies are a valuable source of neuroscientific knowledge; patients are often willing to participate in research while undergoing medical procedures, even though the benefits will probably come to future patients.

The *in vitro* work produces new scientific knowledge but also aims at producing functional neuronal cell crafts for future implant-based treatments (e.g., for brain^6^ and spine^7^). A further research challenge is to train cell crafts *in vitro* or *in vivo* to readily integrate and interact with host tissue. Novel *in vitro* models^ii^ are also used to study neuronal disorders, such as Parkinson's disease^8^ and epilepsy,^9,10^ and for drug development.^11,12^

Considering the linear, nonlinear,^13^ chaotic,^14^ or stochastic^14,15^ systems theory as appropriate for the underlying neuronal system and task at hand, all these tasks would be better explored with closed-loop systems, especially using dialogical paradigms. The brain is a complex system^16^ and probing it with open-loop paradigms has an inherent problem: the same stimulus may result in varying responses. However, using appropriate closed-loop systems, a particular stimulus can be associated with a specific system state, thereby creating a controlled way of exploring the complex system. Thus, closed-loop systems may provide us with novel means of unraveling principles governing complex neuronal system functionality.

Computer simulations (i.e., *in silico*^17^ work) are invaluable in gaining understanding into the workings of neuronal cells,^18,19^ cellular networks,^20–23^ and the brain,^17,24,25^ as well as in drug discovery^26,27^ and understanding disorders of the nervous system.^28^ *In silico* methods are the only possible way to test certain things, such as the effects of EES, taking into account the large biological variance in healthy neurons and disease conditions. *In silico* work is crucial for developing advanced EEM-EES systems, including electroceuticals,^29^ and increasing our understanding of phenomena taking place in neuronal cells, microcircuits, and more extensive neuronal systems due to EES. *In silico* predictions can also be an essential part of closed-loop control. For example, it is still not completely understood why deep brain stimulation (DBS) works (or sometimes does not work); this can be studied, and the stimulation electrodes and paradigms refined *in silico*, including closed-loop EEM-EES systems.^30,31^ When designing more advanced closed-loop EEM-EES systems, it may be advantageous to make the first design phases *in silico*.

### Types and evolution of EES-based systems

EES-based control systems can be categorized into five types based on the intent of the EES and nature of the controller (if any):

Type 1: Open-loop EES systems for causing an effect without feedback. An example of this type of system is DBS^32^ for essential tremor with the DBS always on^33^ and running an *a priori* designed EES paradigm.

Type 2: Closed-loop EEM-EES systems for actuation. An example of this type of system is an EEM-driven robotic arm with tactile force feedback by EES of the person's neuronal system.^34,^^iii^ Type 1 and 2 systems do not, in general, contain explicit controllers. However, neuronal system adaptation may likely still occur.

Type 3: Closed-loop EEM-EES systems for training neuronal systems or for learning. In this type of system, the brain itself acts as the learning controller, and the ICT system, in general, does not contain a controller. For example, BCIs^5^ based on sensorimotor rhythms require that the user learns through perhaps months of training to modify his or her sensorimotor brain rhythm amplitudes to achieve decodable desired responses, which are fed back (e.g., through a display).

Type 4: Closed-loop EEM-EES systems for neuronal system state manipulation. In a type 4 system, the state of the brain or other neuronal system is assessed based on the EEMs, and EES is applied as determined by a controller to change the brain or neuronal system state. For example, DBS can be applied upon automatic detection of essential tremor^35^ to alleviate the tremor or upon a predicted epileptic seizure^36,37^ to prevent the seizure. Type 4 systems can be realized with several different levels of manual or automatic control, as described below. The current challenge is to automatize type 4 systems. Advancing these systems to obtain novel scientific tools and more effective and safer therapeutic methods involves developing better (1) long-term stable EEM-EES electrode systems with spatially known and adaptive electrical measurement and stimulation fields, (2) analysis and understanding of the underlying meaning of EEMs concerning the task at hand, (3) intelligent controllers that take into account the neuronal system states and can anticipate the EES effects and side effects, and (4) adaptive EES paradigms that can selectively affect targeted neuronal circuits and systems and their functionality.

Type 5: Closed-loop EEM-EES systems for meaningful interaction between the ICT and neuronal systems. These systems will be required in the future to fully integrate ICT solutions with neuronal systems to regain lost nervous system functionality, to produce functionally trained neuronal crafts for implant-based therapies, and for new study paradigms to gain insight into information processing in the brain.

**FIG. 1.**
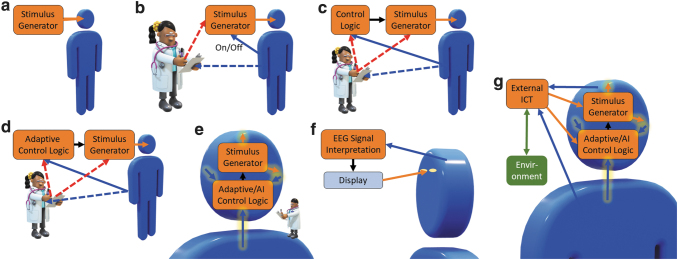
Different technological development stages of stimulator-based medical systems **(a**–**e)** and BCIs **(f**–**g)**. **(a)** An open-loop system in which the stimulator is always on. **(b)** A delayed closed-loop system in which the patient can turn the stimulator on and off, and the doctor can adjust the stimulation parameters. **(c)** A closed-loop system that can measure patient status and automatically turn the stimulator on and off. The doctor can tune the control and stimulus parameters. **(d)** A closed-loop system that can automatically adjust stimulation parameters in addition to the functionality of the system in **(c)**. **(e)** Possible future schema: a fully automated and implanted system that measures several aspects of the patient's status and adapts stimulation to the current situation; the system may also be capable of learning by observing the patient's physiological responses to the adaptive stimulation. The doctor observes possible side effects and safety. Possible health data transfer from the patient to an external ICT system is not depicted. **(f)** A traditional BCI system example: user intent is interpreted from measured EEG signals, for example, and the interpreted intent is shown on screen as feedback to the user. Some EEG-based spellers^42^ have used this approach. **(g)** Possible future schema: an implanted intelligent EEM-EES system in which stimulation is adapted to the state of the highly nonlinear nervous system behavior and user physiology to gain the desired responses. The system is connected to external computing and information resources and the environment with wireless real-time full duplex (i.e., simultaneously bidirectional information transfer). Arrow types: orange, stimulation feeds [in **(f)**, visual]; blue, measured physiological signals (including signals measured from the brain, regardless of the illustrated starting points of the arrows); red, human-mediated parameters; black, system-internal signals; glowing, EEM or EES realized with fully implanted hardware; dashed, signals in delayed loops. AI, artificial intelligence; EEM, extracellular electrical measurement; EES, extracellular electrical stimulation; BCI, brain–computer interface; ICT, information and communications technology; EEG, electroencephalogram.

The widely applied open-loop EEM-EES systems are further contrasted with the emerging closed-loop systems and their different realizations: a clinical open-loop EES system, and different levels of clinical EEM-EES systems are schematically illustrated in Figure 1a–e and two levels of BCIs in Figure 1f and g. Previously, clinical EES was performed in an open-loop manner,^33^ as EES paradigms were designed *a priori*, the stimulator was always on, and there was no feedback (Fig. 1a). After that, delayed manual closed-loop systems (Fig. 1b) offer enhanced controllability compared to open-loop systems as the patient can turn the stimulator on or off, and the doctor can adjust the stimulus generator parameters in an effort to find appropriate stimulus parameters.

The system in Figure 1c observes measurements from the patient, and an automatic controller turns the stimulus generator on and off (e.g., upon detecting symptoms in a bang-bang or on/off controller manner). Such closed-loop EEM-EES systems involve EEM analysis that can detect or predict nervous system malfunction or other relevant events and trigger the EES signal generator accordingly. For this, a robust detectable biomarker^38,39^ for the nervous system malfunction must exist, such as sharp waves in the electroencephalogram (EEG) for some types of epilepsies or electrophysiological surrogates of hand tremor to detect hand usage to alleviate symptoms of Parkinson's only when needed. The biomarkers should reflect the severity of the symptoms^38^ and preferably appear before them so that the EES can prevent the episodes.

At the next level, the EEM-EES system may also be capable of assessing the state of the patient or brain and adapting stimulus parameters accordingly (Fig. 1d).^40^ A doctor would, in general, oversee and possibly tune the system. Finally, the entire system (Fig. 1e) could be implantable and measure and adaptively control the physiological functions to precisely control the disease or symptoms. A doctor could still be involved, for example, to observe possible side effects and for safety.

Examples of BCI system schemas are illustrated in Figure 1f and g; such systems can be of any closed-loop type 2–5 system. In these systems, both the brain and the EEM interpretation system may act as adaptive controllers: the EEM interpretation system may adapt to the measured signals based on system performance, and the person may learn to manipulate his or her physiology to drive the system to achieve a desired response. BCIs have traditionally been closed-loop systems based on measuring physiological signals, interpreting user intent by analyzing the EEMs, and providing feedback on the interpretation to the user^5,41^; Figure 1f illustrates a BCI system based on EEG measurements and visual feedback (e.g., for an EEG-based speller^42^). Such systems have usually not included a controller *per se*, but the closed-loop adaptation has been performed by the user's brain, as noted above.

Also BCI systems utilizing information feedback through the person's neuronal system have been proposed; for example, utilizing EES of a preselected peripheral nerve to cause a useful sensation^34^ or by in-brain connected bidirectional interfaces.^41,43^ Also closed-loop interaction between ICT and the cortex has been proposed.^44^ However, the current systems are types 2–4, and true full-duplex type 5 BCIs have not yet been realized. Type 5 systems, as shown in Figure 1g, would benefit from more involved adaptive or artificial intelligence (AI)-based closed-loop brain stimulation systems^39^ in addition to personal learning.

Open-loop systems illustrated in Figure 1a correspond to type 1 systems. Type 2 systems for actuation can be implemented using any scheme illustrated in Figure 1c, d, f, or g, with the stimulus generator (Fig. 1c, d), display (Fig. 1f), or external ICT (Fig. 1g) replaced or complemented by an actuator. The systems in Figure 1c–g can be used in learning and neuronal system training (type 3 systems) and those in Figure 1c–e and g in neuronal system state manipulation (type 4 systems). The systems in Figure 1f can be used in neuronal system state manipulation; however, EEM interpretation results are not directly utilized to modulate neuronal system state and no EES is applied. Thus, such systems are not type 4.

Given appropriately designed systems, the potential for enhanced therapeutic effects of type 3 and 4 (and in future, type 5) EEM-EES systems can be expected to increase as the technology is advanced through the developmental stages, such as illustrated in Figure 1c–e and g. Finally, type 5 systems could be implemented (Fig. 1g); such BCI systems could find numerous applications in the clinic and perhaps, in the far future, in normal life.

*In vitro* work can produce information on the functioning of neuronal cells and microcircuits at a more refined level than the *in vivo* work (with the exception of small animals with neuronal systems consisting of a very limited number of neurons, such as *Caenorhabditis elegans*^45^). However, in *in vitro*, organism level responses are not present, such as limb movements to sensory inputs or oral descriptions of experiences that could be used for controlling the closed-loop apparatus. The *in vitro* control signals for closed-loop control are measures of electrical neuronal cell activity, such as local field potentials,^46^ AP statistics, network burst statistics,^47^ and network synchronization^48^ estimates. A line of multielectrode EEM analysis methods has been presented by Ylä-Outinen et al.^49^

Stages analogous to the *in vivo* technology stages presented in Figure 1 also exist in the *in vitro* world. *In vitro* EEM-EES systems used in neuroscience research are commonly realized as illustrated in Figure 1b with the patient replaced by *in vitro* cell culture (without the on/off capability), for example, and the doctor replaced by a researcher. Such systems have been successfully used in research for decades to produce a major corpus of neuroscientific information and knowledge.

Closed-loop EEM-EES *in vitro* systems analogous to those in Figure 1c, d, and f (with the display replaced by a microelectrode array [MEA] for direct EES) are also appearing. Examples of such early type 2 and 3 systems are the animats^50^ in which EEMs from an *in vitro* neuronal system grown on an MEA are used to control virtual animal movements, and obstacle detection results are fed back to the *in vitro* neuronal system as EES through the MEA. However, the neuroscientific value of such demonstrations has so far been somewhat limited. After that, *in vitro* closed-loop EEM-EES systems have been on the rise,^4,51,52^ and they can be expected to open up new avenues to study *in vitro* systems as the stimulus can be adapted to the state of the neuronal system, possibly providing controlled access to more input/output states. Furthermore, *in vitro* systems analogous to that illustrated in Figure 1g may offer yet more neuronal system control parameters and outputs and, thus, possibilities for basic neuroscientific findings on *in vitro* neuronal network functioning, and especially with type 5 systems, for meaningful neuro-ICT communications, a prerequisite for bio-ICT convergence.

Similarly, as for *in vivo* and *in vitro*, *in silico* models can be developed for the stages of stimulus paradigms presented in Figure 1. In principle, any system can be simulated *in silico*; however, the level of detail depends on the computational resources and the simulation task at hand. Even if the parameters of the underlying neuronal system are not known, properly designed *in silico* simulators can be used to search the parameter space, for example, by comparing the simulated EEMs to the actual EEMs.^53^ *In silico* modeling can be performed at the levels of (1) molecules^54^ and ion channels,^15^ (2) ionic currents,^55,56^ (3) neuronal cells,^57–59^ (4) neuronal networks,^20,60^ and (5) neuronal systems, such as brain and its regions.^25,28^ Furthermore, the models can be based on biological knowledge or be phenomenological (i.e., aimed at reproducing observed natural phenomena at much lower computational cost than biomimetic models). Despite being such a versatile tool, full closed-loop control of *in silico* neuronal models is still to be exploited.

### Prerequisites for closed-loop EEM-EES systems

A prerequisite for closed-loop EEM-EES schemes to be viable is that control systems must be appropriately faster than the biological processes to be controlled. At the simplest, the EEM-EES loop consists of EEM electrodes, analog filters and amplifiers, analog-to-digital converters, a digital processor (e.g., a personal computer processor, microcontroller, or digital signal processor [DSP]), EES pulse generator/digital-to-analog converters, analog amplifiers, and EES electrodes (which may or may not be the same as the EEM electrodes).

Some lower limits for speed constraints for such closed-loop EEM-EES system operation can be derived from neurobiological parameters so that the detected activity can be effectively reacted upon or intercepted. AP conduction velocities in a nerve fiber are on the order of 0.1–100 m/s,^61^ ^p. 257^ the synaptic latencies in the cortex are 0.2–6 ms,^62^ and the lengths of APs usually seen in our MEA EEMs are ∼2 ms. A common *in vitro* electrophysiology measurement and stimulation system, the MEA2100-System^63^ (Multi Channel Systems MCS GmbH, Reutlingen, Germany), hosts all the necessary components for EEM and EES and an embedded Texas Instruments TMS320C6454^64^ DSP to provide a sub-millisecond closed-loop delay. Depending on the clock speed, The TMS320C6454^64^ is capable of executing 8000 million instructions per second or multiply-accumulate-cycles per second. Thus, at the maximum MEA2100-System EEM sampling rate of 50 kHz (i.e., with 20 μs between the subsequent voltage samples), over 100,000 processor instructions can be executed before new data are available for the DSP. Therefore, closed-loop EEM-EES with simple EEM analysis and closed-loop EES logic can be realized with the MEA2100-System, and the ongoing neuronal activity effectively reacted upon.

To construct *in vitro* EEM-EES systems with more advanced EEM analyses and EES application logics, general-purpose DSPs reaching tens of billions of floating-point operations per second in a single component are available,^65^ and graphics processing units can offer tens or hundreds of billions of floating-point operations per second.^66^ In addition, very fast analog-to-digital and digital-to-analog converters are available. Thus, creating real-time EES-EEM systems that can respond from the neuron's AP point of view “immediately” to the detected neuronal activity, even with more advanced signal analysis or diagnostics and adaptive stimulation delivery using a limited number of electrodes, should not be a problem. However, full-scale analysis and adaptive stimulation of a neuronal system coupled to a large-scale electrode system (e.g., to a 10,000-electrode MEA with a 50 kHz sampling rate and 24-bit sample word length) would require real-time analysis of ∼11 Gb/s, which would still be a challenge.

Other prerequisites for the closed-loop EEM-EES schemes to be viable are that the target system is controllable^67–69^ *per se* and that the selected control systems are theoretically capable of controlling the target systems. Thus, it is crucial to view the (1) neuronal system with its states and state transitions, (2) EEM signals, and (3) controller as linear, nonlinear, chaotic, or stochastic systems, as appropriate. For type 3–5 systems and those in Figure 1c–g (not respectively), the choice of EEM assessment and control methods is greatly affected by the adopted system models. For example, ideally, for a type 4 system for neuronal system state manipulation as illustrated in Figure 1c, there could be two brain states: normal and essential tremor that may be assessed linearly from thalamic activity.^70^ Brain state transition may be viewed as a linear function of EES being turned on or off. If such a bang-bang controller adequately controls the tremor, we do not need to further consider the true nature of the underlying neuronal system.

However, to create more specific and effective control of neuronal systems, we do need to consider that the underlying neuronal systems are nonlinear and chaotic,^13,14^ possibly with stable operational modes. For nonlinear/chaotic systems,^14^ the controllers should be adaptive in EES generation and delivery so that the EES can act on the specific target functionality detected based on the EEMs and affect the target neuronal structures, microcircuits, or single cells in an optimized manner. Fundamentally, the systems must be real-time closed-loop systems, highly preferable as illustrated in Figure 1e.

The above prerequisites may be difficult to fully assess *in vivo* or *in vitro*, for example, due to insufficient capabilities to measure and assess the true nature of the neuronal systems and the large natural variability in health and disease. *In silico* models of neuronal systems at different levels of detail and biological plausibility can be combined with control system models to simulate the effects of EES, including in closed loop.^30,31^

## Introduction to Neuronal Systems and Their Stimulation

### Neurons, neuronal networks, and their observation

**FIG. 2.**
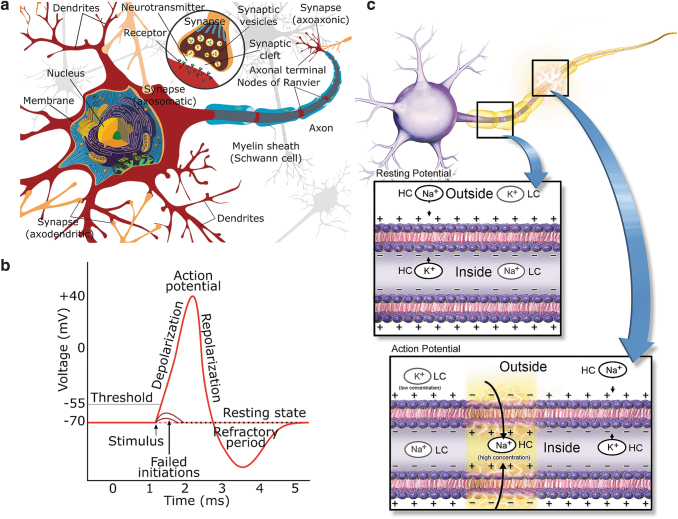
**(a)** An illustration^viii^ of a neuronal cell with synaptic connections to other neuronal cells. A neuron receives inputs through synapses (e.g., to its dendrites from previous neurons in the network and possibly integrates and transmits the received information to subsequent cells using its axon). **(b)** An illustration^ix^ of an AP waveform. In the beginning, the neuron is in the resting state and the cell membrane at resting potential (−70 mV). Upon stimulation, the cell membrane may depolarize, and if the depolarization takes the membrane potential over a threshold, an AP is generated. The AP has a characteristic shape due to orchestrated opening and closing of ion channels in the membrane, as illustrated in **(c)**. **(c)** An illustration^71,^^x^ of a neuron and cell membrane with ion channels in the axon of the neuron, along with indications of relative ion concentrations inside and outside of the cell. Not all different ions participating in an AP are illustrated. At rest, ion concentration differences between the inside and outside of the cell cause membrane resting potential **(b)**. Upon an AP, ion channels open and close in an orchestrated manner allowing different ions to flow in or out so that a propagating AP is created. HC, high concentration; LC, low concentration. AP, action potential.

Figure 2a illustrates a neuron connected to other neurons: inputs from other neurons arrive through the synapses in the dendrites.^iv^ An AP, also called a spike, is the basic unit of information in the nervous system. A neuron may integrate and transmit the information it has received from the preceding neurons through its axon (Fig. 2a) to the subsequent neurons connected to it. When not conveying an AP, the neuronal cell is in the resting state, and ion channels and pumps on the cell membrane maintain a steady negative resting-state membrane potential (Fig. 2b) between the inside and outside of the cell (i.e., across the cell membrane). When an AP reaches a presynaptic terminal of a synapse (Fig. 2a), neurotransmitters are released into the synaptic cleft. An amount of the released neurotransmitters binds to the receptors of the postsynaptic terminal of the receiving neuron, causing the membrane of the neuron to depolarize (i.e., to shift toward zero). If depolarization reaches the AP generation threshold (Fig. 2b), membrane ion channels start to operate in concert (yellow-shaded area in the AP box in Fig. 2c), producing a propagating AP. The characteristic membrane potential waveform due to a propagating AP is illustrated in Figure 2b. During the refractory period (Fig. 2b), the neuron cannot support a new AP. Some types of neurons have axons with myelin sheaths (Fig. 2a). Myelination forms piecewise electrical insulation around an axon, causing the propagating AP effectively to jump from one node of Ranvier to the next (Fig. 2a). An AP propagates faster in a myelinated than in an unmyelinated axon.

A neuronal network consists of at least different types of neuronal^72^ and glial^73^ cells, such as astrocytes and oligodendrocytes. Oligodendrocytes mainly support myelinization (Fig. 2a), but astrocytes have a myriad of functions. They support neurons and their functions in many ways,^74–76^ such as playing a role in memory functions.^77^ Regarding the EES, they play an essential role in information processing at least by modulating synaptic activity by regulating synaptogenesis^78^ and forming a tripartite synapse^20,73^ with the synapses of the pre- and postsynaptic neurons.^73^ Different types of neurons and astrocytes form interactive networks. However, the relations of neurons, astrocytes,^76^ and brain networks and the mechanisms behind synaptogenesis, central nervous system regeneration, and activity-dependent plasticity are not yet fully understood.

The connected cells form local networks or microcircuits, which organize into brain structures, larger brain areas, and finally, the brain. The brain and spinal cord form the central nervous system, which is connected to the peripheral nervous system. The nervous system is also divided into sympathetic (under voluntary control, including the sensory-motor system) and autonomous (including the neuronal systems of the internal organs) nervous systems. Neuronal networks exhibit plasticity,^79,80^ meaning that they are capable of adapting themselves. In other words, the neuronal networks are capable of rewiring themselves due to intrinsic and extrinsic factors, such as electrochemical stimuli or even trauma.

Neuronal cells and their functions are studied with a myriad of tools in molecular and cellular biology^81^ and microscopy.^82^ Directly related to EEM and EES are, for example, calcium imaging^83^ and voltage-sensitive dye microscopy,^84^ since they intrinsically reflect electrical activity. Many of these methods can be combined for the research question at hand; for example, a chemical manipulation can be applied concurrently with EES that may affect the electrical network activity and cellular protein expression. The effects may be observed by microscopy and EEM simultaneously online during the stimulations and later with protein expression analysis. These methods can be applied to *in vitro* neuronal cultures and many *in vivo*. Such phenomena can also be included in computational neuronal cell and network simulations *in silico*.

### Introduction to neuronal cell and system stimulation modalities

In general, “stimulation” is defined as any act on the subject (e.g., a single cell, cell population, or the brain) so that a response is evoked. “A response” is a measurable phenomenon that can take many forms, such as an AP within a few milliseconds after the stimulation. Stimulation may also be “subthreshold,” meaning that no neuronal AP is evoked as an immediate response; however, the stimulation may still elicit a response, such as a chance in the overall electrical activity, cellular network formation and topology, or protein expression. Several modalities can be used to stimulate neuronal cells extracellularly: electrical fields and currents,^85,86^ electromagnetic fields,^87^ chemicals (including gasses^88,89^), light,^90,91^ and mechanical means.^92^ Although the present review is concerned with EES, short descriptions of the noted stimulation modalities are given below since they can all be utilized in constructing closed-loop stimulation-measurement systems for clinical applications and research.

Mechanical stimulability is a functional property of some neuronal cells. In the peripheral nervous system, some nerve endings have specialized structures to transform mechanical stimulation to APs. A part of this information is brought to our consciousness as tactile touch or pain perception, while some remain unconscious, like most subtle muscle and joint stress information. Mechanical stimulation can also have a direct effect on neuronal cells (e.g., ultrasound can be sensed through mechanosensation^93^).

Light stimulation is mostly concerned with the retina and manipulation of neuronal activity using light. Retina and vision research, in general, and the development of clinical methods for retinal disorders naturally often involve light stimulation, which can be combined with electrophysiological recordings.^94^ In optogenetics,^52,90,91^ neuronal cells are genetically modified to express light-sensitive ion channels so that neuronal cells can be activated by light. Light can also be used to silence neuronal activity.^95^

Chemical manipulations include ion channel blockers that alter the basic functioning of the cell; these can be used to study various facets, such as the differences between neuronal cell types, basic cell functionality, and diseases. In addition, manipulating the gas atmosphere of the culture medium, which affects the composition of the medium, can be considered a chemical manipulation. For example, gas atmosphere changes are used in research on the effects of hypo- and hyperoxia.

Electromagnetic^v^ and magnetic fields surround us everywhere, and their biological effects have remained a hot topic due to suspected biological effects of mobile phones and high-voltage power lines. For example, extremely low frequency electromagnetic fields have been noticed to induce neuronal differentiation from stem cells.^87^ In the clinic, transcranial magnetic stimulation (TMS)^96^ has been used, for example, to determine brain areas before surgery and test the functionality of the spine. Electric and magnetic field stimulation are also used to study neuronal networks *in vitro*.^85^

EES of neuronal cells can take place *in vivo*, *in vitro*, or *in silico*. *In vivo* stimulation of human neuronal cells and systems is used in medical settings for therapeutic effects,^3^ such as DBS for Parkinson's disease^97^ and epilepsy^98^ and electric shock therapy for depression^99^ (see also Table 1). *In vivo* animal brain stimulation experiments are conducted, for example, to study brain diseases, find cures, and unravel functions of the brain. Likewise, *in silico*^17^ studies are used to investigate the basic properties,^21,54,57^ functioning,^21^ and networking^20–22^ of neurons and brain areas, as well as diseases,^60^ drug discovery,^54^ and toxicity testing.^100^ *In silico* work also provides a means by which to test various system hypotheses and theories needed for closed-loop control.

**Table 1.** Commercial View of Six Major *in Vivo* Brain Stimulation Methods for Experimental and Clinical Human Use

	Number of ongoing clinical trials^a^	Estimated number of patients treated annually	Annual market size 2018 (million USD)	Success/benefit rate for depression^b^	Examples of manufacturers and companies	Exemplary indications	Exemplary commercial devices and associated web pages
ECT	33	100,000^c,107,108^	14.36^110^	60–77%,^111–113^	Magstim; Halo Neuroscience; Soterix medical; Caputron; The Brain Stimulator; Sooma; Neuroelectrics; neuroCare Group; BrainBox	Increased neuroplasticity	^117–120^
		1,000,000^109^		70–90%^114–116^			
						Fibromyalgia	^121^
tDCS, tES	362	N/A	N/A	18%,^122^		Major depressive disorder and depression	^121,126^
						Chronic pain conditions	^121,126^
				19.9–34%^123–125^			
						Drug-resistant epilepsy	^126^
TMS^d^	350	N/A	883.38^127^	22.6%,^128^ 37.1%,^129^ 63%^111^	NextStim; Magstim; Caputron EB Neuro; DEYMED Diagnostic; MAG and more; Axilum Robotics; BrainBox; Brainsway; Magventure; eNeura; Neuronetics; neuroCare Group; Neurosoft; Soterix Medical; Cloudneuro; Neuronix	Major depressive disorder^e^ and depression^e^	^130–137^
						Psychiatric conditions	^137^
						Migraine^e^	^138^
						Alzheimer's disease	^139^
DBS	155	N/A	831.6–881.9^140,141^	16.0%,^f,142,143^ 24.1%,^f,144^ 29.0%,^145^ 35%,^146^ 50%^147^	Medtronic; Boston Scientific; Deep Brain Innovations; Abbott Laboratories; Aleva Neurotherapeutics; Functional Neuromodulation; Newronika	Epilepsy^e^	^143^
						Parkinson's disease^e^	^148–151^
						Essential tremor symptoms^e^	^148,150,151^
						Alzheimer's disease	^152^
ECoGS, iEEG	≤10	∼180^g,153^	N/A	N/A	NeuroPace	Epilepsy^e^	^154^
VNS	19	>120,000^h,155^	423^155^	20–30%,^156^ 38.9%,^157^ 43.3%^158^	LivaNova; electroCore; tVNS Technologies (Cerbomed); Parasym	Difficult-to-treat depression^e^	^159^
						Chronic/recurrent depression^e^	^159^
						Epilepsy^e^	^160,161^
						Migraine^e^ and cluster headache pain^e^	^162,163^

Global numbers of ongoing clinical trials and treated patients, market sizes, success rates in depression treatment, and examples of manufacturers, indications, and commercially available devices.

This table is indicative only, and the data may vary depending on the source.

^a^ Approximate numbers of active and recruiting clinical trials according to U.S. National Library of Medicine^164^ related to the stimulation methods considered in the table as of May 2020.

^b^ Highly dependent on the disease and study.

^c^ In the United States.

^d^ Including (magnetic) theta-burst stimulation

^e^ United States Food and Drug Administration approved indication.

^f^ Success rates for epilepsy.

^g^ In the United States between 1988 and 2008.

^h^ Altogether by 2018.

DBS, deep brain stimulation; ECoGS, electrocorticogram stimulation; ECT, electroconvulsive therapy; iEEG, intracranial electroencephalography; tDCS, transcranial direct current stimulation; tES, transcranial electric stimulation; TMS, transcranial magnetic stimulation; VNS, vagus nerve stimulation; N/A, not available.

The primary focus of the current review is on EES^86,101^ methods, without completely forgetting closely related magnetic stimulators. Extracellularly applied electric fields impose forces on ions and charged molecules and, thus, induce currents in the extracellular space (and theoretically also intracellular currents and currents across the cellular membrane). The electric currents, in turn, change the ion concentrations and potentials near the neuronal cell membrane. If these phenomena sufficiently depolarize the cell membrane, sodium channels activate, allowing sodium influx. If the resulting membrane depolarization reaches a threshold potential, an AP is generated (Fig. 2b, c). EES affects neuronal cells and networks and may also affect glial cells,^74–76^ such as astrocytes, which support neurons and their functions in many ways.^74–76^ EES is usually delivered in the form of short rectangular voltage or current pulses^51^ or sequences^52^ of pulses. However, neuronal stimulation is a question of stimulation effectiveness and selectivity; in other words, how often a stimulation yields the desired response and how precisely the stimulation affects only the desired tissue or cells.

Electrical manipulation, including EES, may affect neuronal cells and systems in numerous ways^102,103^ and at all levels of the neuronal systems, including the basic properties and functionality of the cells, structural and functional connectivity of the cells and circuits, and the interplay between brain regions. The phenomena that are affected by EES and directly observable by EEM include evoked APs and AP bursts, long-term potentiation or depression,^79^ and altered network behavior due to damage or learning. Appropriate EEM modalities can reveal neuronal electrical activity at the level of single-cell APs (so-called single-unit activity), cell populations, local brain regions, and brain. However, ion channel activity measurements cannot be performed extracellularly, but patch-clamp measurements^104^ of the cell membrane are required.

EES is usually accompanied by measurements; for example, the electrical activity of the neuronal system being stimulated can be measured to observe the pre- and poststimulation states of the neuronal system. EEM *in vivo*^43^ can be performed as EEG measurements on the scalp or as invasive measurements from the cortex or deep structures with implanted electrodes; all of these methods are used both in research and in the clinic. For some *in vivo* applications, such as electric shock therapy or electroconvulsive therapy (ECT; see Table 1), behavior of the subject can be observed. *In vitro*, EEM is generally performed using microelectrodes in varying constellations. *In silico* simulations can be used to generate simulated EEM signals with simulated EES effects.

## Neuronal EES and Magnetic Stimulation *In Vivo*

In the clinic,^105,106^ EES and magnetic stimulation systems are traditionally open loop; however, closed-loop paradigms are emerging, and some have already been approved for clinical use. Most known *in vivo* EES and magnetic stimulation methods are transcranial electrical stimulation, including ECT,^99^ transcranial direct and alternating current stimulation,^103^ TMS,^96^ and DBS.^3,102^ Select market-related statistics and clinical and experimental indications along with exemplary companies and devices for six major EES-based methods for human use are listed in Table 1. It is evident that the methods presented in Table 1 possess potential for numerous ailments and that the global markets are substantial.

ECT, transcranial direct current stimulation, and transcranial alternating current stimulation are administered noninvasively transcranially^165^ using scalp electrodes to feed electric currents through the head to treat various conditions, such as severe or intractable depression, mania, catatonia, or aggression in people with dementia. TMS is applied using electric coils above the scalp, and the peak magnetic field can be aimed at a desired target in the brain using magnetic resonance imaging taken before TMS. TMS is used, for example, to treat dementia and assess the condition of the spine by observing muscle activity evoked by the TMS. TMS can also be used in presurgical brain mapping so that the surgeons know to avoid damaging functionally essential brain tissue.

ECT and TMS are usually open-loop or delayed closed-loop methods in which subsequent treatments are based on clinical poststimulation evaluations. TMS can also be realized in close-loop manner: it can be triggered based on detected events, creating a closed-loop system. For epilepsy control,^166^ TMS upon detection of epilepsy-indicating phenomena in the intracortical EEM signals has been demonstrated to decrease epileptic events.^167,168^

Closed-loop systems have also been proposed. Kraus et al.^169^ studied (in healthy subjects) TMS for neurorehabilitation by applying TMS to induce corticospinal excitation in response to imagined motor activity detected in the EEG. In their experiment,^169^ the controller was of a simple on/off type, triggering a TMS pulse upon observing a predetermined biomarker event-related desynchronization in the EEG β-band (16–22 Hz) during a motor imagery task. Berényi et al.^168^ studied closed-loop TMS for epilepsy control. Again, the controller was a simple bang-bang controller, turning TMS on when so-called spike-and-wave patterns telling of generalized petit mal epilepsy were detected in electrocorticogram (ECoG)^vi^ measurements; the closed-loop TMS was able to shorten the duration of the episodes in rats. All these are based on the idea that a neuronal signal-based feature is used to trigger a predetermined stimulus. This functionality is a leap forward compared to many other clinical EES systems in which the neuronal system status or its implications to the stimulus are not considered.

**FIG. 3.**
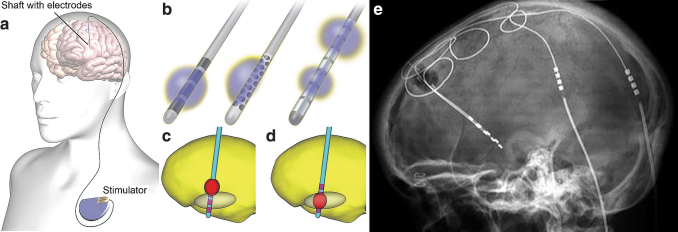
Illustrations of an implanted DBS stimulator, shafts with electrodes, and simulations of stimulated brain regions. **(a)** A schematic illustration^175,^^xi^ of an implanted DBS shaft with electrodes (not visible), leads, and the stimulator (pulse generator). **(b)** Examples of DBS shaft electrodes^176,^^xii^; from left to right, a conventional four-electrode shaft, a multipolar electrode shaft with 40 electrodes for steerable electrical stimulation fields, and an eight-electrode shaft with selectable stimulation electrode configurations, along with exemplary stimulated areas in each case. **(c**, **d)** Visualizations^175,^^xiii^ of a computational model of a four-electrode shaft implanted in the brain with the stimulation target area (oval) and the stimulated brain area (red) in two cases: **(c)** unsuccessfully and **(d)** successfully localized stimulation. **(e)** An X-ray image^xiv^ of a human head with implanted DBS electrodes and leads. Two shafts with electrodes are seen mostly overlapping on the left side of the image and connectors in the two leads on the right. The leads go from the electrode shafts through the skull and continue under the skin to the stimulator implanted in the chest **(a)**. DBS, deep brain stimulation.

DBS (Fig. 3) is realized by implanting stimulation electrodes in deep brain structures for treatment of issues, such as severe and otherwise intractable Parkinson's disease,^170^ essential tremor,^171^ depression,^172^ epilepsy,^37^ obsessive–compulsive disorder,^173^ and pain.^174^ In DBS, usually one or two shafts are inserted into the brain in target structures (Fig. 3a) whose stimulation is clinically known to alleviate symptoms. Each shaft carries one to eight omnidirectional electrodes or a set of electrodes for spatially directed stimulation (Fig. 3b). DBS electrodes are inserted through an opening in scalp, skull, and dura into the brain (Fig. 3a, e) according to a carefully made *a priori* plan, with the patient's head fixed in a stereotactic device. Implantation planning is done based on known stimulation target locations for the particular indication and patient's brain imaging so that major arteries, veins, and other crucial areas will not be harmed. Electrode positioning is usually verified by imaging postimplantation. It is also possible to simulate the brain area to be affected by the stimulation (Fig. 3c, d).

DBS usually operates in a closed-loop manner with delayed manual control; the patient may turn the stimulation on upon perceiving symptoms (Fig. 1b). In addition, automatic closed-loop approaches have been developed for DBS (Fig. 1c). However, for epilepsy, as for any clinical DBS application, the basis of patient selection, electrode positioning in the brain, and EES and EEM parameters are not fully understood.^177^

A proposed approach to essential tremor control^35^ has been to turn on the stimulators when the patient is using the affected limb. Herron et al.^35^ implanted a tremor patient with both an electrode strip on the motor cortex area and DBS electrodes. The DBS was turned on automatically upon detecting hand movement activation through the motor cortex electrodes (Fig. 1c), thus saving DBS battery and alleviating side effects compared to the DBS being on continuously. Usually, however, DBS stimulus adaptation is delayed closed loop since the stimulation paradigm is *a priori* programmed by the doctor and reprogrammed in trial-and-error manner until acceptable treatment response is hopefully achieved (Fig. 1b). Currently, there are no actual type 5 closed-loop stimulus systems that adapt to the state of the complex nervous system.

Vagus nerve stimulation (VNS)^178^ is yet another form of EES used to treat epilepsy^143^ and depression,^178^ and it has been proposed for blood pressure control.^179^ VNS is realized by implanting electrodes around the vagus nerve in the patient's neck^178^ or by external stimulation.^180^ Closed-loop VNS systems using, for example, a controller based on a VNS stimulator parameter state transition model^181^ have been proposed. A handheld noninvasive VNS device previously approved for the treatment of migraine and cluster headaches has also been approved by the U.S. Food and Drug Administration for the duration of the COVID-19 emergency for COVID-19 related asthma-reduced airflow.^182^

**FIG. 4.**
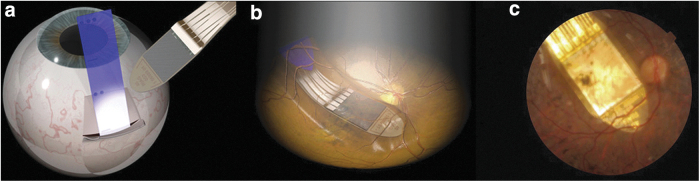
A retinal EES implant^185,^^xv^ to regain sight by EES of retinal ganglion cells. **(a)** Subretinal implantation using a guide foil (blue). **(b)** An illustration and **(c)** fundus photograph of the actual implant postimplantation.

Retinal and visual cortex EES^183^ implants have been developed to regain sight.^184^ For example, retinitis pigmentosa is an inherited disease that leads to the loss of light-sensitive photoreceptors in the retina; however, the retinal ganglion cells and the optic tract to the visual cortex remain intact. A retinal implant may be implanted in the eye under the retina to stimulate retinal ganglion cells to partially regain sight. Edwards et al.^185^ recently reported restoration of partial vision in end-stage retinitis pigmentosa patients by implanting EES devices behind the retinas (Fig. 4) to stimulate ganglion cells at 1600 stimulation sites. In each patient, an implantation was made in the eye with no useful light sensation. The light image to be conveyed by stimulation was detected by the same implanted device, whereas a more conventional approach is to use an external camera. The implant improved the patients' quality of life and also provided objectively measured improvement in object recognition.^185^ Although this system is based on measured incident light and adjusting EES of the inner retina, the system is still open loop. Zrenner^186^ has provided a perspective on the developments in the field, and an excellent review of electrical stimulation of the retina for artificial vision has been presented by Weiland et al.,^187^ discussing the options for MEA locations with respect to the retina, different stimulation target cell types, and a call for the development of smaller stimulation electrodes for effective single-cell stimulation and larger MEAs to provide a larger field of view. One problem in retinal EES is that retinal ganglion cell sensitivity to EES seems to be degreasing with repetitive EES pulse applications^187^; adjustment of the stimulus parameters in a closed loop could provide a solution.

Direct visual cortex EES has also been proposed^188^; however, EES of the visual cortex typically only produces sensations of light blobs, called phosphenes. Rotermund et al.^183^ also proposed direct visual cortex stimulation and hypothesized that adjusting timing and EES parameters in closed loop based on measured visual cortex activity could lead to enhanced visual sensations; however, the system had not yet been tested in practice.

Regarding the above clinical methods, type 5 closed-loop systems remain to be seen despite noninvasive EES having been proposed for rehabilitation from many different ailments, such as for motor function rehabilitation after stroke.^189^ EES has also been shown to enhance neurite growth after spinal cord injury *in vivo*, although functional recovery was not achieved.^190^

Neuroscientific invasive *in vivo* EES-based studies are performed mainly on primates, rodents, and insects in addition to experiments on volunteer patients undergoing clinical brain surgery (e.g., to implant cortical surface electrodes to localize epileptic foci for resective surgery). Invasive *in vivo* EEM and EES methods for neuroscience include ECoG measurement and stimulation (i.e., ECoGS) and intracortical EEM and EES, which have been investigated (e.g., for BCIs^43,191,192^) in search of methods for reading and writing information directly from and to the brain. ECoG measurement and stimulation electrodes can be, for example, macroscopic planar electrodes of a few millimeters in diameter, one or a few centimeters apart, placed on the surface of the cortex under the dura.

For intracortical EEM and EES,^43^ cortex-penetrating electrode shaft assemblies, such as tetrodes, Michigan probes, and Utah arrays,^192^ can be used. To implant such electrodes, the scalp, skull, and dura are opened, and the electrode or, for example, electrode shaft array is lowered into the brain. To achieve sufficient spatial accuracy when implanting electrodes in deep structures (also for DBS), micromanipulators are used with the head of the human or animal fixed in a stereotactic device. The larger planar ECoGS electrodes record and stimulate larger neuronal populations close to the brain surface, whereas spatially more specific EEM and EEM are achieved using the smaller intracortical electrodes. However, the inserted shafts are more damaging than ECoG electrodes on the surface of the cortex.

For basic neuroscience, the mode of operation of EEM and EES systems is essentially open loop. In other words, the stimulus–response effects are observed either electrophysiologically in real time, behaviorally, or by other means, without the EES being modified based on the observations; however, in many occasions, human-in-the-loop does exist (e.g., when searching for adequate EES parameters). That said, closed-loop research is performed in animals to develop human treatments. For example, Salam et al.^193^ compared open-loop and closed-loop EES in the control of chemically induced epileptic seizures in rats. In an online EEM analysis, seizures that were used as the control parameter were predictable on average ∼50 s before seizure onset. EES that started upon a predicted forthcoming seizure was able to reduce the number of seizures by 91%, whereas open-loop stimulation with the same average simulation session frequency reduced seizures only by 19% in the same rats. It would be lucrative to turn such type 4 systems into type 5 systems that would learn to adapt the EES parameters based on the EEMs and seizure prevention history to perhaps achieve even higher performance.

## Neuronal EES *In Vitro*

*In vitro* studies^194^ are conducted using acute brain slices, retinas, and cultured neurons or cocultured neurons and astrocytes, organotypic cultures,^195^ or organoids^196^ (i.e., miniaturized and simplified organs *in vitro*). Extracellular neuronal network function and characterization studies can be done using *in vitro* MEAs,^49,104,197,198^ often utilizing EES. Localized EES using current or voltage pulses or more complex waveforms is usually delivered through microelectrodes with the aim of producing electrophysiological responses. In contrast, electrical and magnetic fields have been used to stimulate *in vitro* neuronal systems, usually without spatial selectivity using static or alternating electric or magnetic fields, such as approximately homogenous fields.

MEA experiments are performed, for example, to probe the electrical responses of cells and networks, alter networks or their activity (e.g., in long-term potentiation and depression experiments^199^) to test neurotoxicity of (potential) drugs and chemicals,^200,201^ or steer stem cell fate and differentiation.^202,203^

**FIG. 5.**
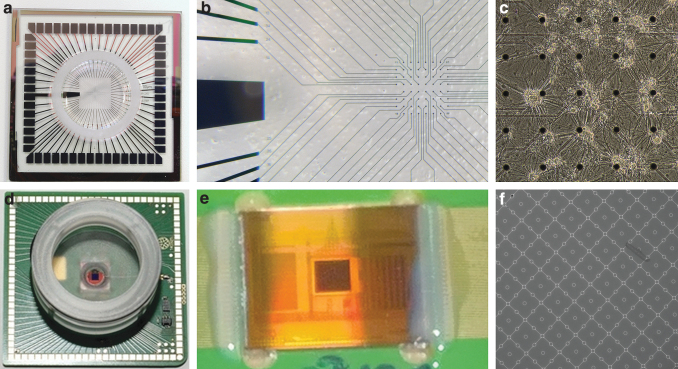
**(a**–**c)** A commercial 60-electrode MEA (MCS): **(a)** a cell culture well and contact pads for connecting the MEA to preamplifiers, **(b)** an MEA (microelectrode diameter, 30 μm; interelectrode distance, 200 μm) and a large electrode, which serves as the ground and reference electrode, and **(c)** a culture of dissociated rat cortical neurons on an MEA. **(d**–**f)** A 4225-electrode complementary metal oxide semiconductor MEA with 1024 stimulation sites (MCS): **(d)** an MEA culture well and contact pads, **(e)** an MEA electrode area (the 2 mm × 2 mm square in the middle), and **(f)** the microelectrodes (small circles, diameter 8 μm; interelectrode distance, 32 μm) and stimulation sites (the square-like areas with single microelectrodes in the middle). MEA, microelectrode array.

*In vitro* MEAs (Fig. 5) can be categorized as passive and active. In passive MEAs (Fig. 5a–c), the electrodes are mere conductors with wiring connecting them to preamplifiers. Usually, the passive MEAs consist of up to 256 microelectrodes^63^ embedded on a planar substrate, such as glass or a printed circuit board. In passive MEAs, the electrode material has galvanic connection to the electrolyte (usually the cell growth medium), enabling charge transfer between the two. Active MEAs (Fig. 5d–f) are usually based on complementary metal oxide semiconductor (CMOS) technology, and the electrodes are parts of active electronic components, such as field-effect transistors. CMOS MEAs may consist of over 10,000^94^ active microelectrodes. The advantage of CMOS electrodes is that they can be made smaller and the electrode density higher than in passive MEAs. Thus, an AP might be measurable using more than one electrode.^94^ However, measurement noise increases with decreasing electrode size

 CMOS MEA electrodes are usually capacitive, which means that the charge transfer on the passivated (coated with a thin insulation layer) CMOS electrode surface is based on capacitive transfer. In contrast, galvanic CMOS MEA electrodes have also been proposed. Recognizing the differences between galvanic and capacitive electrodes is essential for EEM-EES systems since, for example, the stimulus current induced in the culture medium through a capacitive electrode is the derivative of the stimulation voltage.^204^ Similarly, the properties of the galvanic electrodes and their materials affect the stimulus current waveforms as the electrode material–electrolyte interface provides a complex nonlinear response; however, this matter is usually neglected.

A typical MEA system can measure voltages between a reference electrode and each microelectrode (Fig. 5b), for example, at a 5–50 kHz sampling rate with 24 bits/sample. The theoretical analog band of 0–25,000 Hz of such a system is often limited by analog filtering (e.g., 3–3000 Hz). Thus, the systems can capture AP waveforms of ∼2-ms duration with several voltage samples, and if desired, low frequency local field potentials,^vii^ which may also be analyzed separately.

The usual mode of EEM-EES operation with MEA systems is open loop. An *a priori* designed EES is applied, and the effects are observed without modifying the EES based on the observations. Moreover, EEM analysis is also most often performed off line; however, the measurement system is usually able to filter measured data and detect APs and AP bursts and perform some other relatively simple analyses online, allowing the operator to observe the experiment in progress.

Closed-loop EES paradigms^4,197,205,206^ using MEAs have also appeared for bio-ICT interaction, such as robots called animats,^50,207^ controlled by embodied *in vitro* neuronal cultures. Although such experiments have been mostly toy demonstrations of somewhat limited neuroscientific value, they have pushed bio-ICT integration forward. Control of the robot in these systems is a kind of closed loop, but the part of the neuronal system providing the control EEMs is predetermined, usually based on one experimentally determined input/output electrode pair that suits the simple control scheme. Thus, real closed-loop systems with actual neuronal learning or full-duplex type 5 functionality have not yet been realized.

At least one commercially available MEA system, the MEA2100-System^63^ by MCS, contains an embedded DSP (Texas Instruments TMS320C6454) for controlling real-time closed-loop feedback EES. The manufacturer's software can be used to run EES feedback experiments with simple user-defined feedback logic based on the detected APs.^208^ The DSP with access to EEM and EES hardware can be programmed by the user for more advanced feedback logic, other functions, and peripheral input/output. To the best of our knowledge, this functionality remains mostly unexploited. To date, *in vitro* closed-loop MEA systems are using mainly only control strategies corresponding to EEM-EES system types 1–4, and intelligent dialogical type 5 systems are yet to come.

## Neuronal EES *In Silico*

*In silico*^209^ studies are conducted by simulating neuronal cells and networks^21^ in a computer for several purposes noted earlier. They are useful in many ways, from building hypotheses to unraveling functions, some of which are highlighted below. In contrast, computer models of systems are essential for any reasonable control engineering system. *In silico* neuronal simulations can be based on cell biology, cellular networks, or phenomenology. Phenomenological simulations are aimed at reproducing observed natural phenomena without necessarily modeling the underlying biology. In contrast, simulations may be constructed using simple arithmetic (like in certain types of artificial neuronal networks), sets of differential equations (e.g., to simulate evolving neuronal population dynamics), or finite element methods to simulate neuronal cells and simple networks starting with the underlying physics.

Examples related to the current review include simulating the effects of electric fields on neuronal cells. Aberra et al.^210^ simulated three-dimensional neuronal cells, including their electrophysiological behavior and responses to EES. The simulation results corresponded to experimental data, and the simulator allowed the study of the effects of different applied electric fields. The simulator of Popovych et al.^211^ included a network of two neuronal populations whose neuronal cells were simulated starting with ion and synaptic currents; the simulations proposed enhancements to DBS applications related to Parkinson's.

In EES literature, the effects of EES on cells other than neuronal cells are often neglected, which may be due to a lack of knowledge and the great difficulty associated with measuring EES effects, for example, on astrocytes and the network functionality mediated by them. To this end, Lenk et al.^20^ studied the interactions between neuronal and astrocytic networks *in silico*, including EES effects. It could be reasonably straight forward to simulate closed-loop EEM-EES with the *in silico* methods of Lenk et al.^20^ Despite being such a versatile tool, *in silico* systems are still generally types 1–3 and generally do not include simulated adaptively controlled stimulators. In future, type 5 *in silico* systems might be hooked with type 5 physical systems in an effort to decipher the neuronal code.

## Discussion and Conclusions

Most of the closed-loop systems reviewed herein are technologically (but not neurobiologically or regarding materials science) fairly simple. They rely on the detection of specific events and trigger stimulation accordingly, either as defined *a priori* or somewhat adaptively based on the detected events. For example, closed-loop control strategies^212^ proposed for ECoGS-based BCI devices are numerous, including bang-bang controllers, proportional-integral-derivative controllers, artificial neuronal network-based controllers, and support vector machines, among others, similar to any control engineering application.^213,214^ In addition, some more sophisticated closed-loop EES systems, like an ECoGS-based visual cortex-implanted vision system,^183^ have been proposed. However, the vast majority of work reported in the literature on the closed-loop control of neuronal systems has not been approached from proper complex systems or control theory points of view.

One often overlooked aspect of EES-based systems is the stimulus waveform. Most often, EES is delivered as voltage- or current-based mono or biphasic (approximately) square pulses. However, it cannot be known *a priori* if such an EES waveform was the optimum for a given control task. Therefore, Höfling et al.^215^ proposed smooth white noise EES to more closely approximate the physiological signals in the retina. Desai et al.^216^ experimented with different square pulse EES strategies and sinusoidal EES for seizure control in rats; sinusoidal EES was not useful, whereas the effects of theta pulse EES depended on the stimulation timing strategy. Adaptive EES waveforms or patterns^217^ together with adaptive EES electrode constellations to steer the stimulating electromagnetic field may be necessary to tune the EES in closed loop to affect the desired neuronal cells and circuits and their specific functionality or states. Chang and Paydarfar^29^ have noted that for electroceuticals, “The ultimate goal is to one day have these protocols built into the electroceutical system itself, such that the device would find optimal stimulus waveforms, adapting constantly to the patient's responses.^29^” This would be beneficial also for any type 5 systems.

Related to the stimulus pulse discussion, the timing of the stimulus in present closed-loop systems is driven by detected events and not the states of neuronal systems. In a complex system, the same stimulus may cause drastically different responses. Thus, in addition to using the systemic or neural events as triggering parameters, the stimulus (regarding its waveform, site, and timing) should be adapted to the state of the neuronal system. Work on such adaptive systems would eventually provide better control of the systems, improve clinical outcomes, and open new avenues to study complex neuronal systems. To reach this goal, not just bio-ICT interfaces and stimuli but also the dialogical interrogation technologies need to be developed.

With the computing power currently available, dialogical measurement analysis and stimulation paradigms should be very possible; however, the appropriate analysis algorithms and stimulation paradigms are still unknown. MEA technology itself will also likely develop to provide new data on synaptic and ion channel activity and cellular signaling, which may set new requirements on real-time closed-loop neuronal-ICT systems. Novel (perhaps AI-based) analysis algorithms and dedicated ICT solutions are needed to take neuroscience to a new level and further our understanding of the brain. For the design of such methods, exploring nonlinear and chaotic properties and control of neuronal systems is crucial. We believe that the next paradigm shift in neuroscience can be brought about by real-time closed-loop full-duplex neuronal system analysis and bio-ICT interaction paradigms, the dialogical bio-ICT paradigms.

EES and other stimulation modalities are usually supported by a variety of measurement modalities in addition to EEM. The numerous available measurement modalities, such as microscopy, videography, magnetoencephalograms, electromyograms, and chemical, movement, and pressure sensors, provide us with great flexibility to construct future closed-loop measurement and stimulation systems. From the present review, we conclude that neuroscience and clinical applications could benefit significantly from a paradigm shift to adaptive real-time closed-loop analysis/diagnostic and stimulation systems.

## Notes

^i^ EES methods mainly aimed at studying, enhancing, or steering neuronal cell and network growth *in vitro* are excluded.

^ii^ Note that the term “model” is used in two contexts: biological and computational models. For example, a biological model of a human disease could be a condition appearing or induced in, say, rodents *in vivo*, or a disease related property or function manifested in neuronal cells *in vitro*. A computational *in silico* model of the same would be a fully mathematical model constructed in a computer either based on biological and physiological knowledge or phenomena observed in persons, their neuronal networks, or cells with the disease.

^iii^ However, although the prosthetic system^34^ was designed to utilize nerve fibers identified *a priori* for motor control and sensory input, the system setup also included a motor control learning phase in which the subjects learned to control an on-screen cursor by imagined movements conveyed by the electrodes implanted in motor nerves. For this aspect, the system^34^ is type 3.

^iv^ A commonly used simplified view.

^v^ Electromagnetic field frequency ranges: static: 0 Hz, extremely low frequencies: 0–300 Hz, intermediate frequencies: 300 Hz–100 kHz, radio frequencies: 100 kHz–300 GHz. Higher frequencies: infrared light, visible light, ultraviolet light, X-rays, and gamma rays. Other range definitions exist.

^vi^ Electrophysiological signals measured with electrodes inserted into the cortex.

^vii^ Note that in neuroscience, low-frequency MEA signals band-limited to below 100–300 Hz are often called “local field potentials,” whereas APs can be observed at higher frequencies. An MEA signal measured using the 3–3000 Hz bandwidth, for example, is sometimes divided by filtering into local field and action potentials. However, from a purely physics point of view, MEA measurements *per se* are electric field potential measurements regardless of frequency range.

^viii^ An illustration (https://commons.wikimedia.org/wiki/File:Complete_neuron_cell_diagram_en.svg) by “LadyofHats” Mariana Ruiz Villarreal reproduced under public domain. Labels and graphical elements not discussed in this review have been removed from original illustration and the remaining labels edited for readability.

^ix^ An illustration (https://commons.wikimedia.org/wiki/File:Action_potential.svg) reproduced under the Creative Commons Attribution 3.0 Unported license (https://creativecommons.org/licenses/by/3.0/deed.en). The labels and line types of the original illustration have been edited for readability.

^x^ An illustration (https://commons.wikimedia.org/wiki/File:Blausen_0011_ActionPotential_Nerve.png) by Blausen Medical reproduced from^71^ under the Creative Commons Attribution 3.0 Unported license (https://creativecommons.org/licenses/by/3.0/deed.en). The labels of the original illustration have been edited for readability.

^xi^ Illustration from Shamir et al.^175^ (https://commons.wikimedia.org/wiki/File:Typical_deep_brain_stimulation_setup.jpg) reproduced under the Creative Commons Attribution 3.0 Unported license (https://creativecommons.org/licenses/by/3.0/deed.en). Labels of the original illustration have been edited.

^xii^ Illustration from^176^ reproduced under the Creative Commons Attribution License (http://creativecommons.org/licenses/by/4.0/). Labels have been removed from the original illustration.

^xiii^ Illustration from Shamir et al.^175^ (https://commons.wikimedia.org/wiki/File:A_model_for_the_effect_of_electrical_stimulation_in_a_patient%27s_brain.jpg) reproduced under the Creative Commons Attribution 3.0 Unported license (https://creativecommons.org/licenses/by/3.0/deed.en). One panel from the original illustration has been cropped out. Figure panel designators have been edited.

^xiv^ Image by Craig Hacking and Frank Gaillard (https://commons.wikimedia.org/wiki/File:Deep_Brain_Stimulation.jpg) reproduced under the Creative Commons Attribution-Share Alike 4.0 International license (https://creativecommons.org/licenses/by-sa/4.0/deed.en). The original image has been cropped.

^xv^ Image reproduced from Edwards et al.^185^ under the Creative Commons CC-BY license (http://creativecommons.org/licenses/by/4.0/). Shown are three out of the six panels in the original figure, and the panels have been rearranged for presentation herein.
